# AI Machine Learning–Based Diabetes Prediction in Older Adults in South Korea: Cross-Sectional Analysis

**DOI:** 10.2196/57874

**Published:** 2025-01-21

**Authors:** Hocheol Lee, Myung-Bae Park, Young-Joo Won

**Affiliations:** ^1^Department of Health Administration, College of Software and Digital Healthcare Convergence, Yonsei University, Changjogwan, Yonseidae-gil 1, Wonju, 26493, Republic of Korea, +82 (0) 33-760-2257

**Keywords:** diabetes, prediction model, super-aging population, extreme gradient boosting model, geriatrics, older adults, aging, artificial intelligence, machine learning

## Abstract

**Background:**

Diabetes is prevalent in older adults, and machine learning algorithms could help predict diabetes in this population.

**Objective:**

This study determined diabetes risk factors among older adults aged ≥60 years using machine learning algorithms and selected an optimized prediction model.

**Methods:**

This cross-sectional study was conducted on 3084 older adults aged ≥60 years in Seoul from January to November 2023. Data were collected using a mobile app (Gosufit) that measured depression, stress, anxiety, basal metabolic rate, oxygen saturation, heart rate, and average daily step count. Health coordinators recorded data on diabetes, hypertension, hyperlipidemia, chronic obstructive pulmonary disease, percent body fat, and percent muscle. The presence of diabetes was the target variable, with various health indicators as predictors. Machine learning algorithms, including random forest, gradient boosting model, light gradient boosting model, extreme gradient boosting model, and k-nearest neighbors, were employed for analysis. The dataset was split into 70% training and 30% testing sets. Model performance was evaluated using accuracy, precision, recall, F1 score, and area under the curve (AUC). Shapley additive explanations (SHAPs) were used for model interpretability.

**Results:**

Significant predictors of diabetes included hypertension (*χ*²_1_=197.294; *P*<.001), hyperlipidemia (*χ*²_1_=47.671; *P*<.001), age (mean: diabetes group 72.66 years vs nondiabetes group 71.81 years), stress (mean: diabetes group 42.68 vs nondiabetes group 41.47; *t*_3082_=−2.858; *P*=.004), and heart rate (mean: diabetes group 75.05 beats/min vs nondiabetes group 73.14 beats/min; *t*_3082_=−7.948; *P*<.001). The extreme gradient boosting model (XGBM) demonstrated the best performance, with an accuracy of 84.88%, precision of 77.92%, recall of 66.91%, F1 score of 72.00, and AUC of 0.7957. The SHAP analysis of the top-performing XGBM revealed key predictors for diabetes: hypertension, age, percent body fat, heart rate, hyperlipidemia, basal metabolic rate, stress, and oxygen saturation. Hypertension strongly increased diabetes risk, while advanced age and elevated stress levels also showed significant associations. Hyperlipidemia and higher heart rates further heightened diabetes probability. These results highlight the importance and directional impact of specific features in predicting diabetes, providing valuable insights for risk stratification and targeted interventions.

**Conclusions:**

This study focused on modifiable risk factors, providing crucial data for establishing a system for the automated collection of health information and lifelog data from older adults using digital devices at service facilities.

## Introduction

With advancements in medical technology contributing to longer life expectancies, the world is witnessing a rapid acceleration in population aging. The United Nations projects that the global older adult population will increase from 10% in 2022 to 16% by 2025 [1]. This demographic shift significantly drives the increased prevalence of noncommunicable diseases such as diabetes, hypertension, hyperlipidemia, renal failure, arthritis, and Alzheimer disease, which burden the primary health care system [2]. Specifically, the aging population presents challenges in geriatric care within primary health care systems, including shortages of caregiving personnel, financial constraints, and psychological stresses associated with family caregiving.

Among the Organisation for Economic Co-operation and Development (OECD) countries, South Korea is expected to become the first super-aged society—a society where the older adult population accounts for more than 20% of the total population [3]. The rising older adult population and concurrent increase in chronic diseases represent a critical public health issue in Korea. Notably, diabetes is a serious issue, with 39.2% of older adults experiencing diabetes [4]. Diabetes management is crucial, as inadequate control can lead to severe complications, including hypertension and hyperlipidemia [5]. However, the diabetes management rate among the Korean older adult population stands at a mere 30.3%, significantly lower than the awareness rates (84%) and treatment rates (74.8%) [6]. Older adults are particularly vulnerable to diabetes due to aging and consequent physiological changes, as well as lifestyle modifications resulting from physical decline and other medical conditions [7].

The early prevention of diabetes is imperative in older adults. Previous studies have shown that early intervention in older adults can reduce the risk of complications such as cardiovascular diseases, renal failure, and vision impairment [8]. Yet, clinical interventions such as fast blood glucose tests are often needed for diabetes management, and these may not be feasible due to older adults’ physical frailty. Thus, health education and behavioral interventions, not clinical treatments and diagnostics, are vital for preventing diabetes and other chronic conditions in this population [9]. Research indicates that the factors affecting diabetes in older adults differ from those in the general population [10]. While obesity is a risk factor in individuals in their 40s, being underweight elevates the risk for various chronic conditions, including obesity in older adults [11]. The differences in diabetes risk factors for older adults highlight the importance of specific research on the older adult population.

With the global advancement of computer technology, machine learning and deep learning have been used across various fields. Machine learning algorithms can analyze complex and large datasets and identify patterns and risk factors that might not be apparent through traditional statistical methods. In medicine, the potential of machine learning has been demonstrated through an increase in research on disease prediction, personalized medicine, and personalized public health services in clinical and public health care. Particularly, machine learning algorithms can incorporate a broader array of factors and use a wide range of data types to produce generalizable results compared to conventional statistical approaches [12].

However, research using machine learning algorithms to predict diabetes in the Korean older adult population remains sparse. Studies targeting older adults are particularly lacking, partly due to challenges in accessing physical and mental health data, as well as daily lifelog data, due to the mobility constraints of the older population. Thus, a system for the automated collection of health information and lifelog data from older adults must be established using digital devices at service facilities they frequent [10]. This study determines diabetes risk factors among older adults aged ≥60 years by using machine learning algorithms and selects an optimized model. We hypothesize that older adults may be affected by different and identical risk factors compared to younger generations and provide evidence to facilitate policy-making. The findings could serve as a model for other countries with similar demographic changes and health care challenges.

## Methods

### Data Collection

This cross-sectional study was conducted on older adults aged ≥60 years, and the survey was conducted from January to November 2023. Participants aged ≥60 years living in Seoul, South Korea, were recruited among individuals who had participated in the Mind Care Provider Project. Participants in the project voluntarily collected data through public institution promotions aimed at users interested in using health measurement services. To collect data from the participants, a mobile app called Gosufit was developed, which was installed on participants’ digital devices [13].

First, the app measured indicators such as depression, stress, anxiety, basal metabolic rate (BMR), oxygen saturation, heart rate, and average daily step count, and the data were stored on a server. Second, a health coordinator (registered nurse) measured and recorded data on diabetes, hypertension, hyperlipidemia, chronic obstructive pulmonary disease (COPD), percent body fat, and percent muscle. In total, 3674 older adults aged ≥60 years participated in the survey from January 1 to November 30, 2023. After excluding 590 participants due to nonresponse, dropout, or missing data, 3084 participants were included in the final analysis. Nonresponse and dropout occurred when participants did not wish to continue or when data transmission was interrupted due to issues with the app during the survey.

### Instruments

The target variable was the presence of diabetes. Those diagnosed with diabetes by a physician were coded as 1, and those who have not been diagnosed were coded as 0. The predictor variables included hypertension, posttraumatic stress disorder (PTSD), stress, anxiety, depression, BMR, oxygen saturation, average daily step count, hyperlipidemia, COPD, percent body fat, and percent muscle. Hypertension, hyperlipidemia, and COPD were coded as 1 for a physician diagnosis or 0 otherwise. PTSD, stress, and anxiety and depression were assessed using the 5-item PTSD checklist (PCL-5), the short version of the Geriatric Depression Scale (SGDS), and the Hospital Anxiety and Depression Scale (HADS), respectively, with 100-point–based scoring. Oxygen saturation was determined using a digital oximeter per 100% saturation. The average daily step count was measured via the app.

### Statistical Analysis

The Boruta-based feature selection method (FSM) was used for feature selection, which is a wrapper-based FSM that uses the random forest classification algorithm [14]. The entire dataset was randomly divided into a 70% training set and a 30% testing set using a stratified sampling procedure [15].

### Machine Learning Algorithm

The random forest algorithm is an ensemble learning method used for classification and regression. It constructed multiple decision trees during training and output the class or mean prediction (for regression) of individual trees. Random forests corrected overfitting to the training set that is often seen in decision trees. The gradient boosting model (GBM) is a machine learning technique used for regression and classification problems. It generated a predictive model as an ensemble of weak prediction models (typically decision trees). Like other boosting methods, it built the model in a stage-wise fashion and optimized any differentiable loss function to generalize the model. The light gradient boosting model (LGBM) is a gradient-boosting framework that uses a tree-based learning algorithm. It was designed for distributed and efficient operation with a faster training speed, higher efficiency, lower memory use, and better accuracy. The LGBM was capable of processing large-scale data with numerous features and data points while maintaining performance.

The extreme gradient boosting model (XGBM) is an algorithm for tree-based ensemble learning that addresses the slow performance speed and overfitting regularization issues of gradient boosting models. It featured built-in cross-validation and the automatic handling of missing values. The k-nearest neighbors (KNN) model is a simple, versatile, and easy-to-implement supervised machine learning algorithm used for classification and regression. It classified data points based on how their neighbors were classified, stored all available cases, and classified new cases based on a majority vote of its k neighbors. The case assigned to the class was the most common class among the k-nearest neighbors.

### Performance Evaluation Criteria

The performance of the machine learning models was evaluated based on accuracy, precision, recall, F1 score, and area under the curve (AUC). The confusion matrix evaluated classification models by dividing them into positive or negative categories based on the match between actual and predicted classes.

Accuracy represented the proportion of correctly classified data among the total predictions for the hypertension risk group. It measured how accurate the predictions for the hypertension risk group were. The mathematical equation for accuracy is as follows:


$$Accuracy\left(\%\right)=\left(\frac{t_{p}+t_{n}}{t_{p}+t_{n}+f_{p}+f_{n}}\right)\times 100$$

Precision represented the proportion of actual positive samples among the cases that were predicted as positive by the machine learning model. In other words, it indicated the ratio of samples positive for hypertension to those predicted to have hypertension by the model. The mathematical equation for precision is as follows:


$$Precision\left(\%\right)=\left(\frac{t_{p}}{t_{p}+f_{p}}\right)\times 100$$

Recall measured the proportion of those predicted by the machine learning model to have hypertension within the actual hypertension group. It provided the percentage of cases predicted to be at risk for hypertension from the entire hypertension risk group. The mathematical equation is as follows:


$$Recall\left(\%\right)=\left(\frac{t_{p}}{t_{p}+f_{n}}\right)\times 100$$

The F1 score was the harmonic mean of precision and recall. The mathematical equation is as follows:


$$F1score\left(\%\right)=\left(\frac{2t_{p}}{2t_{p}+f_{p}+f_{n}}\right)\times 100$$

The AUC referred to the area under the receiver operating characteristic curve, which was used to evaluate the performance of binary classification models, and an AUC close to 1 indicated better model performance. The AUC equation is as follows:


$$AUC=\int_{X=0}^{1}TPR\left({FPR}^{-1}\left(x\right)\right)dx$$

### Model Interpretability

Shapley additive explanations (SHAPs) interpret the prediction outcomes of machine learning models. SHAPs were introduced by Lundberg and Lee [16] in 2017 and were designed based on a game theory concept known as Shapley values. These values supported prediction interpretation, enabling the assessment of the relative importance among features. Additionally, they helped understand the characteristics with the greatest influence on the model’s predictions, assisting in model improvement or decision-making processes. The mathematical equation is as follows:

$\emptyset_{k}\left(v\right)=\frac{1}{M!}\sum_{S\subseteq M/\left\{k\right\}}\left|S\right|!\left(M-\left|S\right|-1\right)!\left[v\left(S\cup \left\{k\right\}\right)-v\left(S\right)\right]$

### Ethical Considerations

Ethical approval for this study was obtained from Yonsei University Mirae Institutional Review Board (No. 1041849‐202401-SB-021-01), including a supplementary application for expanded data collection. All procedures and data management were conducted following the General Data Protection Regulation and ethical principles outlined in the Helsinki Declaration. Informed consent was obtained from all study participants regarding data collection and the analysis of the data. The questionnaires were submitted entirely anonymously. No form of compensation was provided to the participants.

## Results

### Participant Information

In total, 3084 individuals participated in this study (Table 1). The study population comprised of 895 (29%) individuals with diabetes and 2189 (71%) individuals without. In addition, 1730 (56.1%) had hypertension, and diabetes prevalence significantly differed according to hypertension (*χ*^2^_1_=197.294; *P*<.001). In total, 1803 (58.5%) participants had hyperlipidemia, and diabetes prevalence significantly differed according to hyperlipidemia (*χ*^2^_1_=47.671; *P*<.001). The mean age was 72.66 years in the diabetes group and 71.81 years in the nondiabetes group. The mean stress score was 42.68 in the diabetes group and 41.47 in the nondiabetes group and significantly differed between the two groups (*t*_3082_=−2.858; *P*=.004). The mean heart rate was 75.05 beats/min in the diabetes group and 73.14 beats/min in the nondiabetes group and significantly differed between the two groups (*t*_3082_=−7.948; *P*<.001).

**Table 1.** Respondent characteristics.

Risk factor	Diabetes (n=895)	Nondiabetes (n=2189)	Chi-square or *t* test (*df*)	*P* value
Hypertension, n (%)			197.294^a^ (1)	<.001
No	209 (23.4)	1115 (50.9)		
Yes	686 (76.6)	1074 (49.1)		
Hyperlipidemia, n (%)			47.671^a^ (1)	<.001
No	286 (32)	995 (45.5)		
Yes	609 (68)	1194 (54.5)		
COPD^c^, n (%)			7.764^b^ (3082)	.005
No	856 (95.6)	2135 (97.5)		
Yes	39 (4.4)	54 (2.5)		
Sex, n (%)			3.472^b^ (3082)	.06
Male	60 (6.7)	191 (8.7)		
Female	835 (93.3)	1998 (91.3)		
Age (years), mean (SD)	72.66 (6.31)	71.81 (6.32)	−3.395^b^ (3082)	.001
PTSD^d^, mean (SD)	14.45 (10.02)	14.21 (9.87)	−0.614^b^ (3082)	.5
Stress, mean (SD)	42.68 (9.83)	41.47 (10.97)	−2.858^b^ (3082)	.004
Anxiety, mean (SD)	15.58 (12.09)	15.67 (12.38)	0.179^b^ (3082)	.86
Depression, mean (SD)	19.39 (12.48)	19.42 (12.92)	0.063^b^ (3082)	.95
Percent body fat, mean (SD)	31.00 (8.74)	30.75 (8.66)	–0.727^b^ (3082)	.47
Percent muscle, mean (SD)	35.73 (5.30)	35.95 (5.35)	–0.465^b^ (3082)	.31
BMR^e^, mean (SD)	1192.54 (159.05)	1187.71 (272.54)	–0.496^b^ (3082)	.62
Oxygen saturation (%), mean (SD)	97.12 (3.48)	97.28 (2.44)	1.455^b^ (3082)	.15
Heart rate (beats/min), mean (SD)	75.05 (6.29)	73.14 (5.97)	–7.948^b^ (3082)	<.001
Daily step count, mean (SD)	11789.99 (18061.89)	13710.84 (34980.68)	1.560^b^ (3082)	.12

a Chi-square.

b 1-tailed *t* test.

c COPD: chronic obstructive pulmonary disease.

d PTSD: posttraumatic stress disorder.

e BMR: basal metabolic rate.

### Risk Factor Selection Using Boruta

The importance of features was measured using the Boruta-based FSM (Multimedia Appendix 1). Hypertension, age, percent body fat, heart rate, hyperlipidemia, BMR, stress, and oxygen saturation were identified as important features. These features were included in the machine learning model for diabetes prediction.

### Performance Comparison of Machine Learning Models

**Figure 1.**
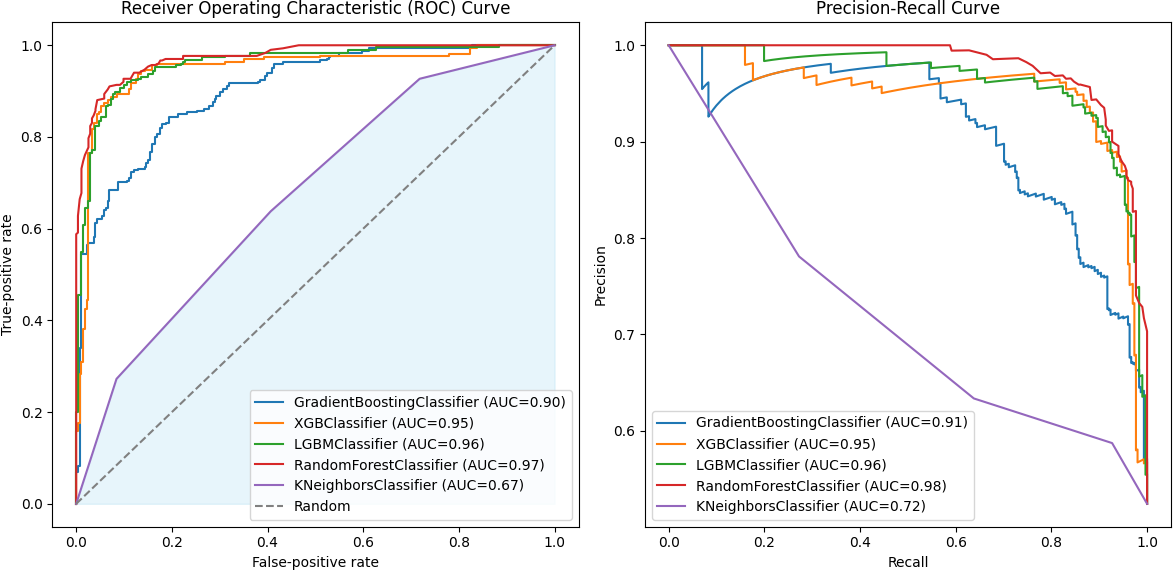
Receiver operating characteristic curves and precision-recall curves of 4 predictive models. AUC: area under the curve; LBGM: light gradient boosting model; ROC: receiver operating characteristic; XBGM: extreme gradient boosting model.

The performances of the 5 machine learning models used in the study were compared based on accuracy, precision, recall, F1 score, and AUC. Model performance was the highest for the XGBM, followed by the LGBM, random forest model, GBM, and KNN model (Table 2, Figure 1). The XGBM had an accuracy of 84.88%, precision of 77.92%, recall of 66.91%, F1 score of 72.00, and AUC of 0.7957, showing a high performance for its prediction.

**Table 2.** Performance of ml methods.

Ranking	Models	Accuracy (%)	Precision (%)	Recall (%)	F1 score	AUC^a^
1	Extreme gradient boosting model	84.88	77.92	66.91	72.00	0.7957
2	Light gradient boosting model	84.77	78.57	65.42	71.39	0.7906
3	Random forest model	81.53	78.82	49.81	61.04	0.7216
4	Gradient boosting model	77.32	77.57	30.85	44.14	0.6360
5	K-nearest neighbors model	66.95	42.06	36.43	39.04	0.5794

a AUC: area under the curve.

### Interpretable Risk Factors of Hypertension

**Figure 2.**
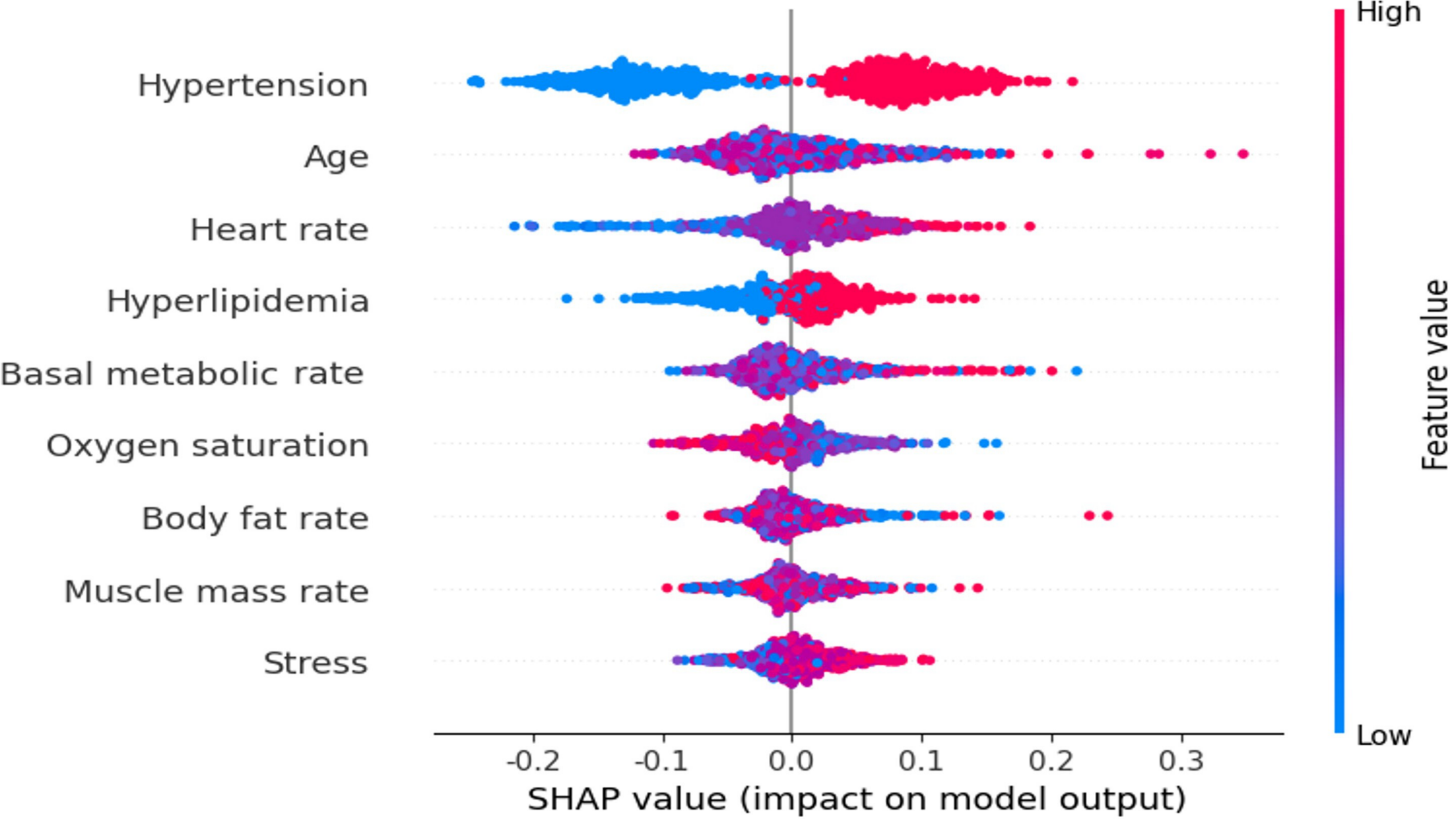
Importance of risk factors based on Shapley additive explanation values. SHAP: Shapley additive explanation.

SHAP analysis was performed for the XGBM—the best-performing diabetes prediction model. The features selected through the FSM (hypertension, age, percent body fat, heart rate, hyperlipidemia, BMR, stress, and oxygen saturation) were included in the SHAP analysis. Red SHAP values indicated a higher impact on diabetes prediction, and blue SHAP values suggested a greater influence on nondiabetes outcomes (Figure 2). The findings indicated positive SHAP values for hypertension, indicating a stronger prediction of having diabetes, while not having hypertension was strongly linked to not having diabetes. Age presented mixed SHAP values, but notably, higher ages correlated with increased diabetes prediction. Similarly, higher heart rates and the presence of hyperlipidemia were associated with increased diabetes risk. Additionally, elevated stress levels were linked to a higher probability of diabetes. The SHAP analysis revealed a mixed impact of age on diabetes prediction. Particularly, as age increased, the likelihood of a diabetes prediction also increased. Additionally, a higher heart rate and the presence of hyperlipidemia were also linked to a higher diabetes probability. Moreover, an increase in stress levels elevated the risk of developing diabetes.

## Discussion

### Principal Results

This study analyzed the performance of 5 machine learning–based algorithms in predicting diabetes risk in older Korean adults. The XGBM performed the best, supporting previous research that shows XGBM as the superior model for diabetes prediction with an AUC of 84% [17], which was also similar to this study. In contrast, other studies have assessed the predictive accuracy of decision trees, naive Bayes algorithms, and random forest algorithms for diabetes risk factors, finding the random forest algorithm to be the top performer with 94% accuracy and precision [18], which was identical to our predictive algorithm. Systematic reviews of machine learning approaches also mention that support vector machines, artificial neural networks, and decision trees are frequently used prediction classification models [19]. However, these were excluded due to the anticipated challenges in managing nonlinear patterns with linear approaches [20].

In this study, hypertension, hyperlipidemia, old age, heart rate, and stress were identified as diabetes risk factors, which was similar to previous reports. Existing studies establish a close link between hypertension and diabetes, with a stronger correlation observed in older adults [21], and this was also seen in our results. A study analyzing diabetes risk factors using large-scale health care data from Kuwait demonstrated that the logistic regression had the highest accuracy at 80.7% and that hypertension, obesity, and sex were also strongly associated with diabetes risk [22]. Further, an increase in heart rate was predicted to elevate diabetes risk. A study of 30,000 participants reported that diabetes risk increased with every 10 beats per minute rise in pulse rate [23], which was consistent with the findings of this study. Hyperlipidemia was also identified as a diabetes risk factor. Hyperlipidemia, hypertension, and diabetes are 3 major chronic conditions that are interrelated as mutual risk factors, and this study confirmed that hyperlipidemia is a diabetes risk factor [24].

This study predicted diabetes in older adults, and certain factors differed from previously known diabetes risk factors. In the existing literature, diabetes risk is elevated with increasing obesity [25], but in this study, the SHAP value for percent body fat was not clear, with no significant differences in diabetes risk according to percent body fat in the independent *t* test. Thus, the severity of obesity was not a significant risk factor for diabetes. On the contrary, diabetes risk increased with decreasing body weight. An array of study results has been reported regarding this issue. One such report suggested that weight loss positively impacts diabetes in older adults but also complicates the treatment for optimal blood glucose regulation in patients with type 2 diabetes [26]. Here, weight loss in older adults entails loss of muscles and bone density, which may have a detrimental impact on diabetes in the long term [27].

Certain predictor variables identified as significant diabetes risk factors in previous studies were not included in this study. Particularly, family history is a known risk factor for diabetes [28]. One important factor of this study was that it focused on modifiable risk factors, rather than family history, which is an unmodifiable risk factor. Particularly, we examined other risk factors besides unmodifiable risk factors, such as family history, age, and sex, as being able to provide interventions for these risk factors is essential. The Korean government should emphasize preventive health education in primary health care facilities, senior welfare centers, community centers, and public health centers to address modifiable diabetes risk factors in the older adult population, such as hypertension, heart rate, hyperlipidemia, and stress. Additionally, while education for older adults is crucial, prevention is more important. Thus, health education should be provided for those in their 40s to prepare for healthy older adulthood.

### Limitations

This study had a few limitations. First, the study population comprised older adults aged ≥60 years who reside in Seoul, Korea. Thus, it did not represent the entire 60-and-over population in the country. Further, while existing studies set the age criterion for older adults as 65 years and older, we set the age to 60 years and older because the retirement age in Korea is 60 years. Second, we could not obtain personally identifiable information such as personal income and education level due to legal regulations. Thus, even though these factors may predict diabetes, this study was limited to other characteristics. Lastly, this study randomly split the dataset into training and testing subsets (70% and 30%). This split was not stratified, which means the distribution of target variables in the subsets may not perfectly match the original dataset’s distribution. This could potentially impact the generalizability of the model’s performance to other datasets. Future studies could consider employing a stratified splitting method to ensure a balanced representation of target variables across subsets.

### Conclusions

This study analyzed machine learning algorithms for diabetes prediction in older adults in Korea. Hypertension, hyperlipidemia, and stress were identified as modifiable diabetes risk factors. Additionally, body fat percentage did not significantly predict diabetes in older adults, presumably because body fat loss is closely linked to muscle strength and bone mass loss. The findings suggest that targeted interventions focusing on managing hypertension, hyperlipidemia, and stress can significantly reduce diabetes risk in this population.

Furthermore, these diabetes predictors in older adults could be mitigated by promoting healthier lifestyle choices and behaviors, such as regular physical activity, balanced nutrition, and stress management techniques. The government should implement comprehensive health education programs across various facilities, including primary health care facilities and welfare centers, to raise awareness about these modifiable risk factors.

Moreover, educational interventions should be initiated at a younger age—particularly for individuals in their 40s—to foster proactive health management and prevent the onset of diabetes in older adulthood. By adopting a preventive approach and addressing modifiable risk factors early, we can enhance the overall health and quality of life for the aging population in Korea. It is necessary to develop personalized modeling that predicts major chronic diseases such as diabetes, hypertension, hyperlipidemia, and obesity through the advancement of prediction algorithms. This will provide a foundation for creating personalized health promotion education and programs. Future research should focus on refining machine learning–based models by incorporating diverse datasets and longitudinal data to improve generalizability and predictive performance. Additionally, exploring the integration of behavioral and environmental factors into machine learning algorithms may further enhance the accuracy and applicability of these models in real-world settings.

## Supplementary material

10.2196/57874 Multimedia Appendix 1 Risk factors selection using the Boruta-based feature selection method.

## Abbreviations

AUC: area under the curve
BMR: basal metabolic rate
COPD: chronic obstructive pulmonary disease
FSM: feature selection method
GBM: gradient boosting model
HADS: Hospital Anxiety and Depression Scale
KNN: k-nearest neighbors
LGBM: light gradient boosting model
OECD: Organisation for Economic Co-operation and Development
PCL-5: 5-item PTSD checklist
SGDS: short version of the Geriatric Depression Scale
SHAP: Shapley additive explanation
XGBM: extreme gradient boosting model
