# Supplementary figures and images for: AI Machine Learning–Based Diabetes Prediction in Older Adults in South Korea: Cross-Sectional Analysis
Source: JMIR Form Res. 2025 Jan 21;9:e57874. doi: 10.2196/57874 (PMC11779598; doi:10.2196/57874)

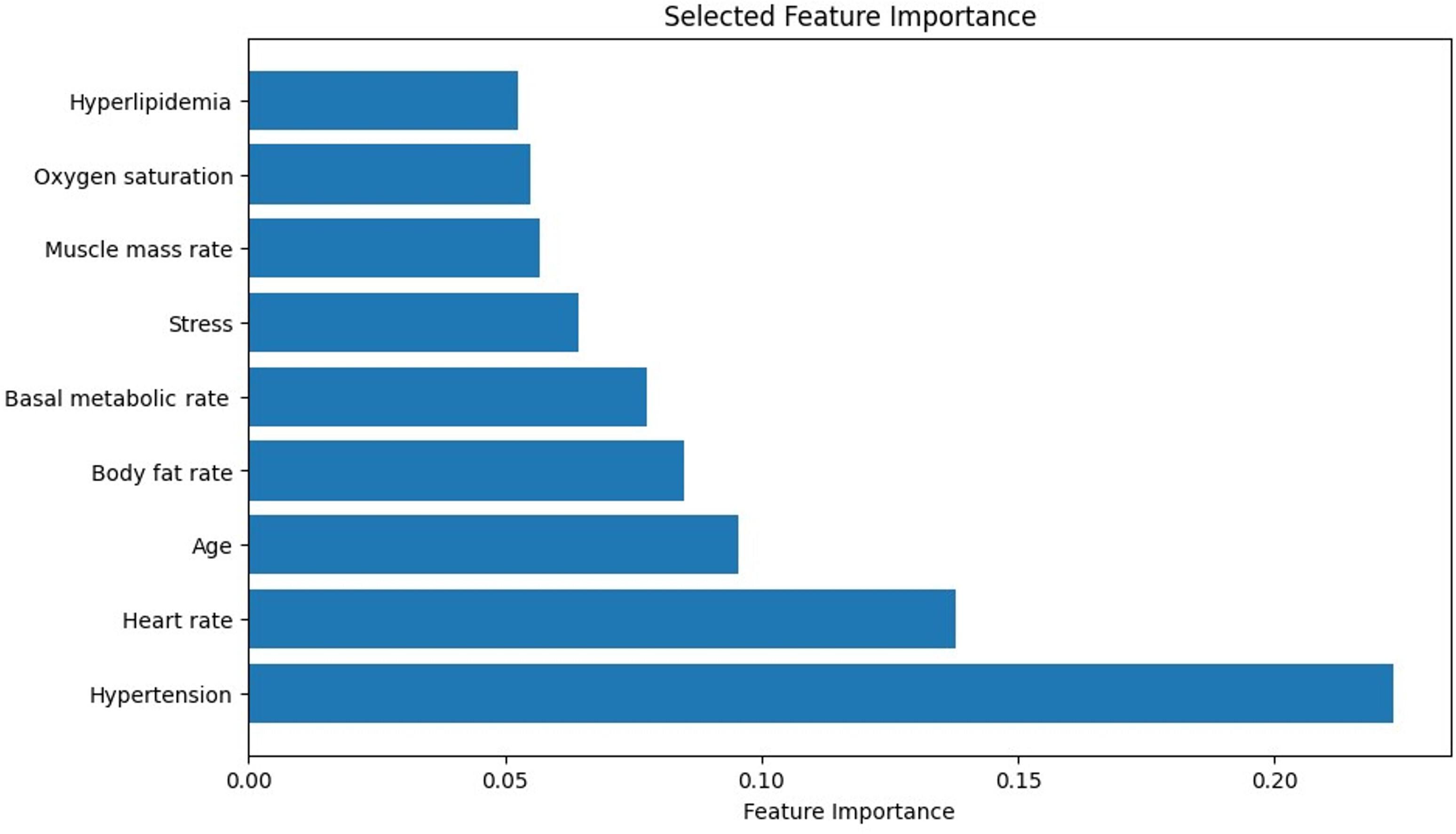

Supplement: Multimedia Appendix 1 [file formative-v9-e57874-s001.jpeg]
